# Quality assessment of scientific outputs using the BWM

**DOI:** 10.1007/s11192-017-2284-3

**Published:** 2017-02-16

**Authors:** Negin Salimi

**Affiliations:** Leiden University, Leiden, The Netherlands

**Keywords:** Research evaluation, Scientific outputs, Quality metrics, Multi-criteria decision-making, BWM

## Abstract

Assessing the quality of scientific outputs (i.e. research papers, books and reports) is a challenging issue. Although in practice, the basic quality of scientific outputs is evaluated by committees/peers (peer review) who have general knowledge and competencies. However, their assessment might not comprehensively consider different dimensions of the quality of the scientific outputs. Hence, there is a requirement to evaluate scientific outputs based on some other metrics which cover more aspects of quality after publishing, which is the aim of this study. To reach this aim, first different quality metrics are identified through an extensive literature review. Then a recently developed multi-criteria methodology (best worst method) is used to find the importance of each quality metric. Finally, based on the importance of each quality metric and the data which are collected from Scopus, the quality of research papers published by the members of a university faculty is measured. The proposed model in this paper provides the opportunity to measure quality of research papers not only by considering different aspects of quality, but also by considering the importance of each quality metric. The proposed model can be used for assessing other scientific outputs as well.

## Introduction

With increasing the number of scientific outputs, assessing quality of them has become difficult. Assessment of quality of scientific outputs is vital and crucial issue not only for researchers themselves but also in a higher level, for heads of university sections, managers of research institutes, and even research funding agencies. At one hand, researchers have faced with competitive situation in which even high quality proposals may not always guarantee funding or tenure. On the other hand, several key decisions in university depend on the assessment of scientific outputs of researchers such as decisions on employment of a new staff member, promotion of current staff members or granting of scientific awards (Costas et al. 2010).

There are different types of scientific outputs such as scientific papers, conference papers, books, reports, databases, and slides. In this study, we aim to assess the quality of a research paper although the proposed model can be extended to evaluate other scientific outputs. Although determining the quality of each paper is a subjective issue and depends on the person who assesses the quality of paper, but it is necessary to follow a structured framework including some objective metrics as policy makers, research managers, and even scientists need objective metrics to make decisions on quality of papers. This is in line with what Gratch (1985) mentioned in his study. He cited: “to assess the quality of research paper bibliographies, criteria and a process for rating must be formulated”. To be precise, each paper should have a basic quality comprises methodology quality (refers to accuracy of used methods and techniques), cognitive quality (refers to the content of scientific ideas), and esthetic quality (refers to the attractiveness of models and mathematical formulations) to be published. This assessment is called scientific peer review (Benos et al. 2007). Peer review/peer assessment is subjective and it is done by peers/qualified experts (Moed et al. 1985). The evaluation of basic quality is necessary for each paper to be published (Moed 2006; Taylor 2011). However, because it is a subjective measure, it has biases (Benos et al. 2007; Moed 2007; Zahedi et al. 2014). More importantly, peer review is not sufficient to evaluate the quality of a paper, especially after the paper is published, as it is a partial quality indicator (Martin and Irvine 1983). Hence, it is necessary to measure the quality of research papers with other metrics. Citation analysis has been used for assessing impact of a research paper in existing literature (Judge et al. 2007; Moed 2006). In other words, citation analysis is considered as a tool to measure the “scientific impact” of research paper as well as peer review (Moed 2006). Yet, using the two metrics peer review and citation analysis is not sufficient to fully evaluate “scientific impact” of papers. We rather think that to evaluate a paper we should consider other aspects of quality such as educational impact (refers to the usability of the research paper as a course syllabus for undergraduate and graduate students) and scientific collaboration impact (refers to the number of nationally and internationally co-authored scholarly outputs) of the research paper.

Some other metrics such as usage data analysis (Duy and Vaughan 2006; Rowlands and Nicholas 2007), social media metrics/altmetrics (Priem and Hemminger 2010; Zahedi et al. 2014), web citations and link analysis (webometrics) (Thelwall 2008) have been used as a single metric to evaluate research in existing literature of research evaluation. However, using a single metric to measure the quality of a research paper provides a limited view of quality (Zahedi et al. 2014). The necessity of research assessment considering multiple metrics/criteria is remarked by Ronald and Fred (2013) and Zahedi et al. (2014). In other words, to understand and evaluate quality of the research paper we need more metrics than relying merely on a single metric. Yet, there is not any systematic study that investigates the quality of a research paper by considering a set of metrics to cover all aspects of quality.

As such, the main aims of this study are:

1. to formulate some objective metrics for measuring quality of a peer reviewed paper.
2. to measure the quality of research papers using a multi-criteria methodology—best worst method (BWM), which is used to find the importance of metrics. One of the salient features of the proposed assessment method is its generalizability. That is to say, quality of other scientific outputs such as reports, slides, blog posts, books, and conference proceedings can be assessed through the proposed assessment method.

The remainder of this paper is organized as follows. In Sect. 2, we review existing literature to find proper metrics to assess the quality of a research paper. In Sect. 3, we propose a methodology to identify the weights/importance of identified metrics for quality assessment of papers. In Sect. 4, we present our empirical analysis and discuss the findings. The paper ends with the conclusions, implications and future research directions in Sect. 5.

## Literature on research evaluation

Publication and citation analysis, two bibliometric indicators, have been used in the literature as very popular research evaluation tools (Noyons et al. 1999; Zahedi et al. 2014). In fact, these bibliometric indicators are used to assess the research activities in several studies such as Costas et al. (2010), King (1987) and Moed et al. (1985, 1995). Moreover, bibliometric indicators are identified as objective and useful research evaluation tools at different levels of analysis. This levels are in continuum from macro level (countries), meso level (regions, areas, and centers) to micro level (research teams and individual researchers) (Noyons et al. 1999).

In macro and meso levels, university rankings provide a useful tool to compare universities in national and international levels to each other based on different bibliometric indicators. The Leiden Ranking is a global university ranking based on bibliometric indicators including publication output, citation impact (comprising mean citation score, proportion top 10% publications), and scientific collaboration (comprising proportion collaborative publications, proportion international collaborative publications, mean geographical collaboration distance, and proportion long distance collaborative publications) (Waltman et al. 2012).There are other commonly used global university rankings such as Academic Ranking of World Universities (ARWU),^1^ Times Higher Education World University Rankings (THE),^2^ and Scimago Institutions Rankings (SIR).^3^

Snowball metrics^4^ also provide a standard for universities to understand their strengths and weaknesses and consequently to improve their strategies. Snowball metrics comprise input, process and outcome metrics. Metrics in input level refer mainly to the volume of research grant applications, volume of awards granted, private investment leveraged from public sponsorship and volume of business engagements. In process level, metrics are mainly on volume of research income spent, percentage of sector total research income per institution, and total value of contract research. Finally, in outcome level, the metrics are: publications and citations, scholarly output, citation count, citations per output, h-index, field-weighted citation impact, outputs in top percentiles, publications in top journal percentiles, collaboration, collaboration impact, academic-corporate collaboration, academic-corporate collaboration impact, altmetrics, public engagement, intellectual property volume, intellectual property income, sustainable spin-offs, and spin-off-related finances.

In micro level, bibliometric indicators are used to analysis different issues such as measuring performance of collaborative Ph.D. candidates compared to the non-collaborative Ph.D. candidates (Salimi et al. 2015), assessment of research performance (Costas et al. 2010; Moed et al. 1985, 1995), and using web for research evaluation (Vaughan and Shaw 2005; Moed et al. 1995).

Costas et al. (2010) measured the research performance of scientists considering more dimensions. They introduced three main bibliometric dimensions for measuring the research performance of scientists as follows: (1) observed impact dimension including the percentage of highly cited papers, the internationally normalized impact and the citations per publication (2) journal quality dimension including the median Impact Factor, the normalized journal position, and the journal citation score mean/field citation score mean, and (3) production dimension including the total number of publications, the total number of citations, and the h-index.

In the literature, citation analysis is considered not only as a metric to measure the research performance of scientists (micro level), and to measure the performance of university (meso level), but also it has been used to measure the scientific impact of research papers (see, for instance, Moed (2006, 2009)). However, this metric is not able to measure all aspects of quality of a research paper (Bornmann and Leydesdorff 2013). As accumulation of citations takes time, we see citation one/two years after publishing or even longer (Priem et al. 2012). Therefore, it is limited to evaluate the real-time quality of the research papers. Also, citation count cannot be used for measuring quality of other documents (i.e. slides, reports, databases), which have different format from peer reviewed papers and conference proceedings (Priem et al. 2012). Due to the limitation and weakness of citation count, other metrics such as usage data analysis (Duy and Vaughan 2006; Rowlands and Nicholas 2007), social media metrics/altmetrics (Priem and Hemminger 2010; Zahedi et al. 2014), and web citations and link analysis (webometrics) (Thelwall 2008) have been used as a single metric to evaluate research outputs. However, to the best of our knowledge, a complete evaluation using different metrics has not yet been studied. In following sub-section by combining the research evaluation metrics, which have been used in existing literature and Scopus database, we provide different objective metrics to cover the most important aspects of quality.

### Metrics to assess quality of research papers

Scopus from Elsevier, Google Scholar from Google, and Web of Science from Thompson Scientific are three databases for citation tracking (Bakkalbasi et al. 2006). In addition to these three databases Falagas et al. (2008) studied PubMed database and, by doing comparison study among these four databases, found that Scopus database covers more journals and its citation analysis is faster than other databases. Scopus database^5^ has used some metrics which cover both the impact of and community engagement with a paper. For citation metrics, it uses citation counts, filed-weighted citation impact (its source is snowball metrics), and citation benchmarking. Engagement metrics include four altmetrics dimensions (its source is snowball metrics): scholarly activity, social activity, scholarly commentary, and mass media. More detailed information with their definition is presented in Table 1, which is adopted from Scopus website.

**Table 1:** Article metrics detail (Source: Scopus website)

Metric	Definition
*Citation metrics*	
Citation count	Citation count shows how many times a publication has been cited
Field-weighted citation impact^a^	Field-weighted citation impact is the ratio of the total citations actually received by the denominator’s output, and the total citations that would be expected based on the average of the subject field
Citation benchmarking^b^	Citation benchmarking shows how citations received by this paper compare with the average for similar papers. 99th percentile is high, and indicates a paper in the top 1% globally
*Engagement metrics*	
Scholarly activity	Scholarly activity indicates the number of times a paper has been posted to online tools that are typically used by academic scholars, such as Mendeley
Social activity	Social activity indicates the number of times a paper has stimulated social media posting on platforms used by the general public, such as in Twitter and Facebook
Scholarly commentary	Scholarly commentary indicates the number of times a paper has been commented upon in online tools typically used by academic scholars, such as science blogs and Faculty of 1000 reviews
Mass media	Mass media indicates the number of times a paper has been referred to in publicly distributed news channels

^a^A field-weighted citation impact of: exactly 1 means that the output performs just as expected for the global average. More than 1 means that the output is more cited than expected according to the global average; for example, 1.48 means 48% more cited than expected. Less than 1 means that the output is cited less than expected according to the global average

^b^Citation benchmarking takes into account: the date of publication, the document type (if there are enough papers), and disciplines associated with its source. Citation benchmarking compares papers within an 18 month window and is computed separately for each of its sources’ disciplines. A minimum set of 2500 similar papers is required. The citation benchmark card only appears when compared to all three criteria. Citation benchmarks compared to only discipline and age, but not document type, appears in the Benchmarking section of the citations tab if insufficient data is available to compare on all three criteria

In the following section, we explain all metrics which cover most aspects of quality of research outputs.

#### Citation metrics

Citation analysis has been used extensively for research evaluation (Zahedi et al. 2014). Moed et al. (1985) distinguished between short-term and long-term impact. Citation count is a measure of short-term impact of each paper from publishing time to few years after publishing while long-term impact refers to the durability of publication which can be determined after a very long time. The latter impact is not interested of university science policy makers who concerned about evaluation of current research.

Based on Snowball metrics, field-weighted citation impact considers the differences in research behavior across disciplines as in some fields such as medicine and biochemistry, researchers produce more publications with more citations while in some others such as mathematics or social sciences we see less publications with fewer citations. In fact, this comes from discipline differences, not from performance differences. Therefore, field-weighted citation impact considering disciplinary differences provides more accurate base to evaluate the scientific quality of research papers which is more meaningful than relying only on citation count. In addition to considering citation count along with field-weighted citation impact, Scopus database focuses on how citations received by a specific paper compare with the average for similar papers (with the same age and document type) by using citation benchmarking. Therefore, these three citation metrics (citation count, field-weighted citation impact, and citation benchmarking) together give more complete picture of scientific quality by covering more aspects of scientific impact.

#### Engagement metrics

Social media metrics or altmetrics are new metrics to measure broader aspects of research impact in social web (Priem et al. 2012). In fact altmetrics measure the quality of scientific outputs considering online/social aspects of them. These online/social aspects can be tracked by several online tools through online activities such as tweeting and sharing on Facebook, bookmarking on Delicious, citing on Wikipedia, and linking tweets on Tweeter (Priem et al. 2012). Snowball metrics identified four altmetrics: scholarly activity, social activity, scholarly commentary, and mass media. Scholarly and social activity refer to the number of times that scientific scholars have posted scientific outputs through online tools usually used by scientific scholars (e.g. CiteULike, Mendeley) and in social media (e.g. Facebook, Tweeter, LinkedIn, and Google +) respectively. Moreover, these online metrics contain the number of times that scientific scholars have commented on scientific outputs in online tools that are mostly used by scientific scholars (e.g. science blogs, video posts such as those on YouTube, vimeo, peer reviews such as Publons and Wikipedia). This online metric is called scholarly commentary. Finally the number of times that a scientific output has been referred to by press clippings and news websites (e.g. the Guardian) refers to mass media.

These four altmetrics provide useful information especially for researchers to find out their strengths and weaknesses with regard to their online/social activities.

#### Scientific collaboration metrics

Increasing scientific collaboration, has led to increase co-authorship networks among scientists (Kretschmer 2004). The structure of scientific collaboration by focusing on co-authorship networks has been investigated in several studies (Abbasi et al. 2011; Glänzel and Schubert 2005; Kretschmer 2004; Li et al. 2013; Liu et al. 2005; Newman 2001). Co-authorship networks are a sign of knowledge sharing activity of authors which leads to creation of knowledge (Stokols et al. 2005). In other words, in scientific collaboration, several valuable resources such as information and knowledge are shared to provide social capital for the collaborators (Li et al. 2013). It has been shown that social capital positively impacts on knowledge creation (McFadyen and Cannella 2004), knowledge transferring (Walter et al. 2007) and knowledge contribution (Wasko and Faraj 2005). Co-authorship networks can be studied in different levels of national versus international, single-discipline versus cross-discipline collaboration and institutions (Mattsson et al. 2008). In Snowball metrics, not only the proportion of scientific outputs co-authored by researchers from academia in national and international levels is considered, but also scholarly outputs co-authored by researchers from both academic and industrial affiliations in national and international levels are considered.

#### Educational metrics

Research and teaching are two main activities in universities (Jensen 1988). The relation between scientists’ research and teaching in higher education is a topic which has been studied in the literature (see, for instance, Griffiths (2004) and Jenkins et al. (2007)). In other words, the extent that scientists’ researches improve the teaching and student learning is one of the important concerns of universities. Several studies, by conducting survey as well as interviews among university faculty have found that academics, through their research, add value to teaching and student learning (see, for instance, Jensen (1988) and Smeby (1998)). More precisely, research papers recommended by lecturers for their students to read, have educational impact. Smeby (1998) found that the positive relationship between research and teaching is stronger at graduate rather than undergraduate level. Therefore, one another aspect of quality of scientific outputs is their educational utility. Educational value of a research paper refers to the extent that the research paper is used in a course syllabus for undergraduate and graduate students.

In sum, the quality metrics which are identified for measuring quality of research papers in this study are as follows:

1. *Citation metrics* include citation count, field-weighted citation impact, and citation benchmarking.
2. *Engagement metrics* include scholarly activity, social activity, scholarly commentary, and mass media.
3. *Scientific collaboration metrics* include national versus international collaboration, single-discipline versus cross-discipline collaboration, and academia versus industrial affiliation collaboration.
4. *Educational metrics* include usability of the research paper for undergraduate students and usability of the research paper for graduate students.

As can be seen, there are four main quality metrics with some sub-metrics. As such, the problem of scientific output assessment can be nicely formulated as a multi-criteria decision analysis, where there are four main criteria and some sub-criteria per each main criterion. Formulating the problem this way, we need to use a multi-criteria methodology to find the weights of the criteria and sub-criteria in order to assess the quality of a research output as an integrated score. By doing this a decision/policy-maker (faculty dean in a university) would be able to calculate one aggregated score for each paper which is comparable to the other papers published by the faculty members of the same faculty.

**Fig. 1:**
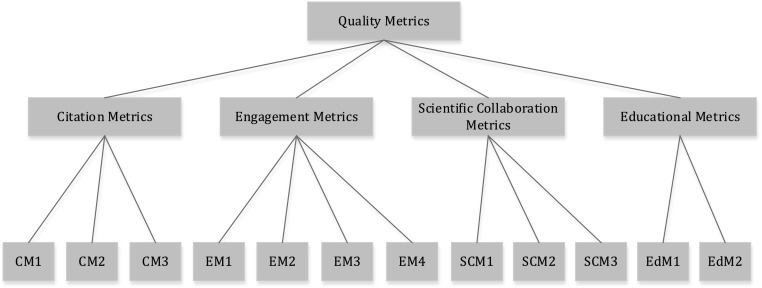
A conceptual model. CM1: citation count, CM2: field-weighted citation impact, CM3: citation benchmarking; EM1: scholarly activity, EM2: social activity, EM3: scholarly commentary, EM4: mass media; SCM1: national versus international collaboration, SCM2: single-discipline versus cross-discipline collaboration, SCM3: academia versus industrial affiliation collaboration; EdM1: usability of the research paper for undergraduate students, EdM2: usability of the research paper for graduate students

Figure 1 shows a conceptual model composing of all metrics to measure quality of research papers.

In the following section, we describe the methodology we used to evaluate the quality of research papers.

## Methodology

### Measuring different weight using BWM

As mentioned before the assessment of scientific outputs can be formulated as a multicriteria decision analysis for which we should use a multi-criteria decision analysis method. There are several multi-criteria decision analysis methods (see, for instance, Greco et al. 2010). In this paper we use a new method called best worst method (BWM) (Rezaei 2015, 2016). We use the BWM as it requires less comparison data and produces more reliable results. In other words, BWM makes the judgment easier and more understandable for decision makers and finally results in more consistent comparisons. The BWM has been successfully applied in different assessment studies such as supplier assessment (Rezaei et al. 2015, 2016), Ph.D. efficiency assessment (Salimi and Rezaei 2016), risk assessment (Torabi et al. 2016), of technological innovation assessment (Gupta and Barua 2016), among others. We describe the steps of BWM to calculate the weights for quality metrics (main criteria and sub-criteria) as follows (Rezaei 2015, 2016):

**Step 1** Determine a set of quality metrics.

In this step, we identify *m* metrics {*I*_1_, *I*_2_, …, *I*_m_} used for evaluation of a research paper.

**Step 2** Determine the best (e.g. most desirable, most important) and the worst (e.g. least desirable, least important) metric according to the decision-maker perspective (in this study our decision-makers are authors and head of section).

**Step 3** Determine the preference of the best metric over all the other metrics, using a number between 1 and 9 (1: *i* is equally important to *j*; 9: *i* is extremely more important than *j*). The result is a best-to-others (BO) vector:

$$A_{B} = (a_{B1} ,a_{B2} , \ldots ,a_{Bm} )$$

where *a*_*Bj*_ indicates the preference of the best metric *B* over metric *j* and *a*_*BB*_ = 1.

**Step 4** Determine the preference of all the metrics over the worst metric, using a number between 1 and 9, which results in the others-to-worst (OW) vector:

$$A_{W} = (a_{1W} ,a_{2W} , \ldots ,a_{nm} )^{T}$$

where *a*_*jW*_ indicates the preference of the metric *j* over the worst metric *W* and *a*_*WW*_ = 1.

**Step 5** Find the optimal weights $(w_{1}^{*} ,w_{2}^{*} , \ldots w_{n}^{*} )$.

The aim is to determine the optimal weights of the metrics, such that the maximum absolute differences $\left\{ {\left| {w_{B} - a_{Bj} w_{j} } \right|,\left| {w_{j} - a_{jW} w_{W} } \right|} \right\}$ for all *j* is minimized, which is translated to the following minmax model:

1 $$\begin{aligned} & \hbox{min} \,\mathop {\hbox{max} }\limits_{j} \left\{ {\left| {w_{B} - a_{Bj} w_{j} } \right|,\left| {w_{j} - a_{jW} w_{W} } \right|} \right\} \\ & {\text{s}}.{\text{t}}. \\ & \sum\limits_{j} {w_{j} = 1} \\ & w_{j} \ge 0,\quad {\text{for}}\,{\text{all}}\;\;j \\ \end{aligned}$$

Problem (1) is transferred to the following linear problem:

2 $$\begin{aligned} & \hbox{min} \,\xi^{L} \\ & {\text{s}}.{\text{t}}. \\ & \left| {w_{B} - a_{Bj} w_{j} } \right| \le \xi^{L} ,\quad {\text{for}}\,{\text{all}}\;\;j \\ & \left| {w_{j} - a_{jW} w_{W} } \right| \le \xi^{L} ,\quad {\text{for}}\,{\text{all}}\;\;j \\ & \sum\limits_{j} {w_{j} = 1} \\ & w_{j} \ge 0,\quad {\text{for}}\,{\text{all}}\;\;j \\ \end{aligned}$$

Solving problem (2), the optimal weights $(w_{1}^{*} ,w_{2}^{*} , \ldots w_{n}^{*} )$ and $\xi^{L*}$ are obtained.

$\xi^{L*}$ is considered as a consistency index. That is, the closer the value of $\xi^{L*}$ to zero, the higher the level of consistency of the comparisons.

## Results and analysis

### Weights of quality metrics

In order to find the weights of the identified quality metrics (criteria) in Sect. 2, we interviewed dean of Technology, Policy and Management faculty in Delft University of Technology, collecting comparison data needed for BWM. Table 2, shows the weights of the four main criteria (citation, engagement, scientific collaboration and educational metrics) and their items (sub-criteria) based on data which are gathered from the dean.

**Table 2:** Relative weight of the criteria and sub-criteria

Criteria	Criteria weights	Sub-criteria	Local weights of sub-criteria	Global weight of sub-criteria^a^
Citation metrics	0.45	Citation count	0.17	0.08
		Field-weighted citation impact	0.54	0.25
		Citation benchmarking	0.29	0.13
Engagement metrics	0.27	Scholarly activity	0.47	0.13
		Social activity	0.26	0.07
		Scholarly commentary	0.17	0.05
		Mass media	0.11	0.03
Scientific collaboration metrics	0.18	National versus international collaboration	0.17	0.03
		Single-discipline versus cross-discipline collaboration	0.54	0.10
		Academia versus industrial affiliation collaboration	0.29	0.05
Educational metrics	0.09	Usability of the research paper for undergraduate students	0.33	0.03
		Usability of the research paper for graduate students	0.67	0.06

^**a**^The global weights of a sub-criterion is obtained by multiplying the local weight of that sub-criterion by the weight of the main criterion to which it belongs. For instance the global weights of “citation count” in obtained by multiplying the weight of “citation metrics” (0.45) by the local weight of “citation count” (0.17) or: 0.45 × 0.17 = 0.08

As can be seen from Table 2, Column 2, *Citation metrics* is the most important quality metrics (0.45), followed by *Engagement metrics* (0.27) *Scientific Collaboration Metrics* (0.18), and *Educational metrics* (0.09) according to the judgment of the dean.

As can be seen from the fourth column of Table 2, among three sub-criteria of citation metrics, *field*-*weighted citation impact* is the most important item. *Scholarly activity* is also the most important item of engagement metrics. For the scientific collaboration metrics, the most important item is *single*-*discipline versus cross*-*discipline collaboration.* Finally, among two educational metrics, *usability of the research paper for graduate students* is more important than *usability of the research paper for undergraduate students*.

The global weights of the sub-criteria are reported in Table 2, Column 5. As can be seen from this column, from among all the sub-criteria *Field*-*weighted citation impact* (0.25), *Citation benchmarking* (0.13) and *Scholarly activity* (0.13) are the most important sub-criteria, together accounting for more than 50% of the importance of the entire quality assessment of a scientific paper.

### Quality item-scores of 54 research papers

By gathering data of quality items of 54 research papers of two sections^6^ of Technology, Policy and Management faculty at Delft University of Technology, through Scopus, we got the quality item-scores of these research papers (see Table 3). The data source for all quality metrics except educational metrics is Scopus and we only considered the research papers (neither conference papers nor book chapters) which are published in 2012. To gather data of usability of research papers in both graduate and undergraduate levels (educational metrics) we asked the authors about their papers by email.

**Table 3:** Quality item-scores of 54 research papers

Paper no.	Citation metrics			Engagement metrics				Scientific collaboration metrics			Educational metrics	
	C1	C2	C3	En1	En2	En3	En4	S1	S2	S3	Ed1	Ed2
1	0.08	0.15	0.82	0.05	0.00^a^	0.00	0.00	1.00	0.00	0.00	0.00	0.00
2	0.08	0.18	0.95	0.41	0.00	0.00	0.00	0.00	0.00	0.00	0.00	0.00
3	0.07	0.25	0.89	0.09	0.00	0.00	0.00	0.33	0.00	0.00	0.00	0.00
4	0.01	0.05	0.56	0.09	0.00	0.00	0.00	0.00	0.00	1.00	0.00	0.00
5	0.04	0.11	0.77	0.57	0.00	0.00	0.00	0.00	0.00	0.00	0.00	0.00
6	0.03	0.08	0.67	0.33	0.00	0.00	0.00	0.33	0.00	0.00	0.00	0.00
7	0.00	0.00	0.00	0.14	0.00	0.00	0.00	0.67	0.50	0.00	0.00	0.00
8	0.05	0.08	0.35	0.14	0.00	0.00	0.00	1.00	0.50	0.00	0.00	0.00
9	0.08	0.15	0.82	0.05	0.00	0.00	0.00	1.00	0.00	0.00	0.00	0.00
10	0.19	0.41	0.89	0.43	0.00	0.00	0.00	0.00	0.00	0.00	0.00	0.00
11	0.07	0.16	0.73	0.42	0.00	0.00	0.00	0.00	0.00	1.00	0.00	0.00
12	0.15	0.36	0.86	0.45	0.00	0.00	0.00	0.00	0.00	0.00	0.00	0.00
13	0.13	0.34	0.90	0.08	0.00	0.00	0.00	0.33	0.00	0.00	0.00	0.00
14	0.05	0.18	0.79	0.17	0.00	0.00	0.00	0.00	0.00	0.00	0.00	0.00
15	0.21	0.52	0.95	0.43	0.00	0.00	0.00	0.00	0.00	1.00	0.00	0.00
16	0.07	0.29	0.77	0.20	0.00	0.00	0.00	0.33	0.00	0.00	0.00	1.00
17	0.15	0.36	0.86	0.45	0.00	0.00	0.00	0.00	0.00	0.00	0.00	1.00
18	0.00	0.00	0.00	0.09	0.00	0.00	0.00	0.00	0.00	0.00	0.00	0.00
19	0.08	0.16	0.85	0.09	0.00	0.00	0.00	0.00	0.00	0.00	0.00	0.00
20	0.09	0.21	0.92	0.14	0.00	0.00	0.00	0.00	0.00	0.00	0.00	1.00
21	0.17	0.38	0.87	0.13	0.00	0.00	0.00	0.00	0.00	0.00	0.00	0.00
22	0.00	0.00	0.00	0.00	0.00	0.00	0.00	0.00	0.00	0.00	0.00	0.00
23	0.23	0.75	0.93	0.13	0.00	0.00	0.00	0.67	0.50	0.00	0.00	1.00
24	0.04	0.10	0.61	0.03	0.00	0.00	0.00	0.33	0.50	0.00	0.00	0.00
25	0.12	0.29	0.00	0.11	0.00	0.00	0.00	0.00	0.00	0.00	0.00	0.00
26	0.29	0.43	0.99	0.08	0.00	0.00	0.00	0.00	0.00	0.00	0.00	0.00
27	0.31	0.65	0.92	0.64	0.00	0.00	0.00	0.00	0.50	0.00	1.00	1.00
28	0.13	0.30	0.75	0.28	0.00	0.00	0.00	0.33	0.00	0.00	1.00	1.00
29	0.08	0.16	0.85	0.09	0.00	0.00	0.00	0.00	0.00	0.00	1.00	1.00
30	0.03	0.09	0.71	0.11	0.00	0.00	0.00	0.00	0.00	0.00	0.00	0.00
31	0.13	0.43	0.95	0.45	1.00	0.00	0.00	0.33	0.00	0.00	1.00	0.00
32	0.16	0.52	0.96	0.09	0.00	0.00	0.00	0.33	0.00	0.00	1.00	0.00
33	0.20	0.48	0.90	0.05	0.00	0.00	0.00	0.33	0.00	0.00	0.00	0.00
34	0.37	0.68	1.00	1.00	0.00	0.00	0.00	0.00	0.50	0.00	1.00	1.00
35	0.05	0.14	0.51	0.13	0.00	0.00	0.00	0.00	0.00	0.00	0.00	0.00
36	0.11	0.34	0.90	0.28	0.00	0.00	0.00	0.00	1.00	0.00	1.00	0.00
37	0.23	0.84	0.96	0.39	0.00	0.00	0.00	0.00	0.00	0.00	1.00	1.00
38	0.00	0.00	0.00	0.00	0.00	0.00	0.00	0.33	0.50	0.00	0.00	0.00
39	0.28	0.60	0.97	0.63	0.00	0.00	0.00	0.00	0.00	0.00	0.00	1.00
40	0.07	0.17	0.65	0.07	0.00	0.00	0.00	0.00	0.50	0.00	0.00	0.00
41	0.07	0.20	0.00	0.09	0.00	0.00	0.00	0.00	1.00	0.00	1.00	0.00
42	0.16	0.25	0.96	0.34	0.00	0.00	0.00	0.00	0.50	0.00	1.00	1.00
43	0.03	0.09	0.71	0.11	0.00	0.00	0.00	0.00	0.00	0.00	0.00	0.00
44	0.13	0.43	0.95	0.45	1.00	0.00	0.00	0.33	0.00	0.00	0.00	0.00
45	0.16	0.52	0.96	0.09	0.00	0.00	0.00	0.33	0.00	0.00	0.00	0.00
46	1.00	1.00	0.99	0.51	0.00	0.00	0.00	0.00	0.00	0.00	0.00	0.00
47	0.03	0.08	0.00	0.08	0.00	0.00	0.00	0.00	0.00	0.00	0.00	0.00
48	0.28	0.60	0.97	0.63	0.00	0.00	0.00	0.00	0.00	0.00	0.00	0.00
49	0.01	0.04	0.51	0.09	0.00	0.00	0.00	0.00	0.00	0.00	0.00	0.00
50	0.29	0.67	0.97	0.21	0.00	0.00	0.00	0.33	0.50	0.00	0.00	0.00
51	0.19	0.41	0.89	0.43	0.00	0.00	0.00	0.00	0.00	0.00	0.00	0.00
52	0.19	0.63	0.95	0.07	0.00	0.00	0.00	0.00	0.00	0.00	1.00	0.00
53	0.15	0.36	0.86	0.45	0.00	0.00	0.00	0.00	0.00	0.00	0.00	0.00
54	0.08	0.16	0.85	0.09	0.00	0.00	0.00	0.00	0.00	0.00	0.00	0.00

We normalized the data which are gathered from Scopus

C1: citation count; C2: field-weighted citation impact; C3: citation benchmarking; En1: scholarly activity; En2: social activity; En3: scholarly commentary; En4: mass media; S1: national versus international collaboration; s2: Single-discipline versus cross-discipline collaboration; S3: academia versus industrial affiliation collaboration; Ed1: usability of the research paper for undergraduate students; Ed2: usability of the research paper for graduate students. Research papers with number 1–17 belong to the Economics of Technology and Innovation section and the reset (papers 18–54) are from section Transport and logistics

^a^We have found several papers with zero engagement activities, as this is a quite new area for sharing scientific results

### Measuring quality of research papers based on weights of different quality items

In this section, we aim to measure quality of 54 research papers using quality item-scores and the weights of quality items. Table 4, contains the quality of each paper based on the items (sub-criteria) of each criterion. Furthermore, the overall aggregate amount of quality for each research paper based on items of all criteria and overall rank of all research papers based on this aggregate number are shown in Table 4, Column 14 and 15 respectively.

**Table 4:** Quality of 54 research papers

Paper no.	Citation metrics			Engagement metrics				Scientific collaboration metrics			Educational metrics		Agg. overall	Rank overall
	C1	C2	C3	En1	En2	En3	En4	S1	S2	S3	Ed1	Ed2		
1	0.01	0.04	0.11	0.01	0.00	0.00	0.00	0.03	0.00	0.00	0.00	0.00	0.19	36
2	0.01	0.04	0.13	0.05	0.00	0.00	0.00	0.00	0.00	0.00	0.00	0.00	0.23	31
3	0.01	0.06	0.12	0.01	0.00	0.00	0.00	0.01	0.00	0.00	0.00	0.00	0.21	32
4	0.00	0.01	0.07	0.01	0.00	0.00	0.00	0.00	0.00	0.05	0.00	0.00	0.15	44
5	0.00	0.03	0.10	0.07	0.00	0.00	0.00	0.00	0.00	0.00	0.00	0.00	0.20	33
6	0.00	0.02	0.09	0.04	0.00	0.00	0.00	0.01	0.00	0.00	0.00	0.00	0.16	43
7	0.00	0.00	0.00	0.02	0.00	0.00	0.00	0.02	0.05	0.00	0.00	0.00	0.09	50
8	0.00	0.02	0.05	0.02	0.00	0.00	0.00	0.03	0.05	0.00	0.00	0.00	0.17	42
9	0.01	0.04	0.11	0.01	0.00	0.00	0.00	0.03	0.00	0.00	0.00	0.00	0.19	37
10	0.01	0.10	0.12	0.06	0.00	0.00	0.00	0.00	0.00	0.00	0.00	0.00	0.29	19
11	0.01	0.04	0.10	0.05	0.00	0.00	0.00	0.00	0.00	0.05	0.00	0.00	0.25	28
12	0.01	0.09	0.11	0.06	0.00	0.00	0.00	0.00	0.00	0.00	0.00	0.00	0.27	22
13	0.01	0.08	0.12	0.01	0.00	0.00	0.00	0.01	0.00	0.00	0.00	0.00	0.23	30
14	0.00	0.05	0.10	0.02	0.00	0.00	0.00	0.00	0.00	0.00	0.00	0.00	0.18	38
15	0.02	0.13	0.13	0.06	0.00	0.00	0.00	0.00	0.00	0.05	0.00	0.00	0.38	11
16	0.01	0.07	0.10	0.03	0.00	0.00	0.00	0.01	0.00	0.00	0.00	0.06	0.27	21
17	0.01	0.09	0.11	0.06	0.00	0.00	0.00	0.00	0.00	0.00	0.00	0.06	0.33	15
18	0.00	0.00	0.00	0.01	0.00	0.00	0.00	0.00	0.00	0.00	0.00	0.00	0.01	53
19	0.01	0.04	0.11	0.01	0.00	0.00	0.00	0.00	0.00	0.00	0.00	0.00	0.17	39
20	0.01	0.05	0.12	0.02	0.00	0.00	0.00	0.00	0.00	0.00	0.00	0.06	0.26	27
21	0.01	0.09	0.12	0.02	0.00	0.00	0.00	0.00	0.00	0.00	0.00	0.00	0.24	29
22	0.00	0.00	0.00	0.00	0.00	0.00	0.00	0.00	0.00	0.00	0.00	0.00	0.00	54
23	0.02	0.18	0.12	0.02	0.00	0.00	0.00	0.02	0.05	0.00	0.00	0.06	0.47	5
24	0.00	0.02	0.08	0.00	0.00	0.00	0.00	0.01	0.05	0.00	0.00	0.00	0.17	41
25	0.01	0.07	0.00	0.01	0.00	0.00	0.00	0.00	0.00	0.00	0.00	0.00	0.09	48
26	0.02	0.11	0.13	0.01	0.00	0.00	0.00	0.00	0.00	0.00	0.00	0.00	0.27	25
27	0.02	0.16	0.12	0.08	0.00	0.00	0.00	0.00	0.05	0.00	0.03	0.06	0.53	2
28	0.01	0.07	0.10	0.04	0.00	0.00	0.00	0.01	0.00	0.00	0.03	0.06	0.32	17
29	0.01	0.04	0.11	0.01	0.00	0.00	0.00	0.00	0.00	0.00	0.03	0.06	0.26	26
30	0.00	0.02	0.09	0.01	0.00	0.00	0.00	0.00	0.00	0.00	0.00	0.00	0.13	45
31	0.01	0.11	0.13	0.06	0.07	0.00	0.00	0.01	0.00	0.00	0.03	0.00	0.41	7
32	0.01	0.13	0.13	0.01	0.00	0.00	0.00	0.01	0.00	0.00	0.03	0.00	0.32	16
33	0.02	0.12	0.12	0.01	0.00	0.00	0.00	0.01	0.00	0.00	0.00	0.00	0.27	24
34	0.03	0.17	0.13	0.13	0.00	0.00	0.00	0.00	0.05	0.00	0.03	0.06	0.60	1
35	0.00	0.03	0.07	0.02	0.00	0.00	0.00	0.00	0.00	0.00	0.00	0.00	0.12	47
36	0.01	0.08	0.12	0.04	0.00	0.00	0.00	0.00	0.10	0.00	0.03	0.00	0.38	13
37	0.02	0.21	0.13	0.05	0.00	0.00	0.00	0.00	0.00	0.00	0.03	0.06	0.49	4
38	0.00	0.00	0.00	0.00	0.00	0.00	0.00	0.01	0.05	0.00	0.00	0.00	0.06	51
39	0.02	0.15	0.13	0.08	0.00	0.00	0.00	0.00	0.00	0.00	0.00	0.06	0.44	6
40	0.01	0.04	0.09	0.01	0.00	0.00	0.00	0.00	0.05	0.00	0.00	0.00	0.19	35
41	0.01	0.05	0.00	0.01	0.00	0.00	0.00	0.00	0.10	0.00	0.03	0.00	0.20	34
42	0.01	0.06	0.13	0.04	0.00	0.00	0.00	0.00	0.05	0.00	0.03	0.06	0.38	9
43	0.00	0.02	0.09	0.01	0.00	0.00	0.00	0.00	0.00	0.00	0.00	0.00	0.13	46
44	0.01	0.11	0.13	0.06	0.07	0.00	0.00	0.01	0.00	0.00	0.00	0.00	0.38	10
45	0.01	0.13	0.13	0.01	0.00	0.00	0.00	0.01	0.00	0.00	0.00	0.00	0.29	18
46	0.08	0.25	0.13	0.07	0.00	0.00	0.00	0.00	0.00	0.00	0.00	0.00	0.52	3
47	0.00	0.02	0.00	0.01	0.00	0.00	0.00	0.00	0.00	0.00	0.00	0.00	0.03	52
48	0.02	0.15	0.13	0.08	0.00	0.00	0.00	0.00	0.00	0.00	0.00	0.00	0.38	12
49	0.00	0.01	0.07	0.01	0.00	0.00	0.00	0.00	0.00	0.00	0.00	0.00	0.09	49
50	0.02	0.17	0.13	0.03	0.00	0.00	0.00	0.01	0.05	0.00	0.00	0.00	0.40	8
51	0.01	0.10	0.12	0.06	0.00	0.00	0.00	0.00	0.00	0.00	0.00	0.00	0.29	20
52	0.01	0.16	0.13	0.01	0.00	0.00	0.00	0.00	0.00	0.00	0.03	0.00	0.33	14
53	0.01	0.09	0.11	0.06	0.00	0.00	0.00	0.00	0.00	0.00	0.00	0.00	0.27	23
54	0.01	0.04	0.11	0.01	0.00	0.00	0.00	0.00	0.00	0.00	0.00	0.00	0.17	40

C1: citation count; C2: field-weighted citation impact; C3: citation benchmarking; En1: scholarly activity; En2: social activity; En3: scholarly commentary; En4: mass media; S1: national versus international collaboration; S2: single-discipline versus cross-discipline collaboration; S3: academia versus industrial affiliation collaboration; Ed1: usability of the research paper for undergraduate students; Ed2: usability of the research paper for graduate students

Among these 54 papers, paper 34 has the first place in terms of quality (total quality: 0.60), while paper 22 has the least quality (total quality: 0.00). Data which is presented in Table 4 provides information regarding the quality of each paper based on each quality metrics and moreover based on different items of each quality metrics. Therefore, this provides us to compare papers based on their detailed quality features. For instance, paper 15 (from Economics of Technology and Innovation section) has the higher quality based on citation and scientific collaboration metrics compared to paper 28 (from Transport and Logistic section). However, paper 28 has a better quality in terms of educational aspects compared to paper 15. Focusing deeply on papers based on the items of each quality metrics shows us that, for instance paper 25 from Transport and Logistic section has a higher quality in terms of citation count and field-weighted citation impact compared to paper 24 from Transport and Logistic section. While paper 24 has a higher quality only regarding citation benchmarking.

The information in Table 4, provides us to not only compare the quality of papers in one section based on different quality metrics and the items of each quality metrics, but also we can compare the quality of papers in two different sections.

### The association of research papers’ quality with Impact Factor of journal

As we mentioned, peer review for assessing the quality of a research paper is not enough as peer review is done by peers who do not necessarily have specific knowledge to assess the quality (Seglen 1997). Impact factor (IF) of journal is one option for evaluation of research outputs. In existing literature, IF has been used to measure the quality of journal (see, for instance, Inayatullah and Fitzgerald (1996)), to evaluate individual researchers and research groups to promote scientists and to allocate research funding (Seglen 1997). However, IF for research evaluation has some serious drawbacks, for instance, IF depends on the research field (journals with high IF are more likely to cover more basic research), and journal IF dose not determine the article citations but article citation rates determine the journal IF (Seglen 1997). Therefore, we think that IF of journal is a kind of criterion to measure the quality of journal instead of a criterion for measuring the quality of a research paper. In fact, scientific scholars contribute to journals through citation as citation influences the impact factor of journal. Therefore, we did not consider IF as one quality metric of scientific outputs in our study. Nevertheless, to understand more, we investigate the relationship between quality of research papers and the IF of journal. The question that arises here is whether there are trade-offs in terms of journal IF and total quality which are represented by citation, engagement, scientific collaboration and educational metrics. We found the IF of the journals that our sample (54 papers) have been published in. We then calculated the correlation between the journal IFs and the aggregated quality of the papers (Table 4, column 14). The results show a low correlation (0.338, sig = 0.013), which means that journal IF is not a proper representative for quality of a research paper which is in line with the previous findings (e.g. Seglen 1997).

**Fig. 2:**
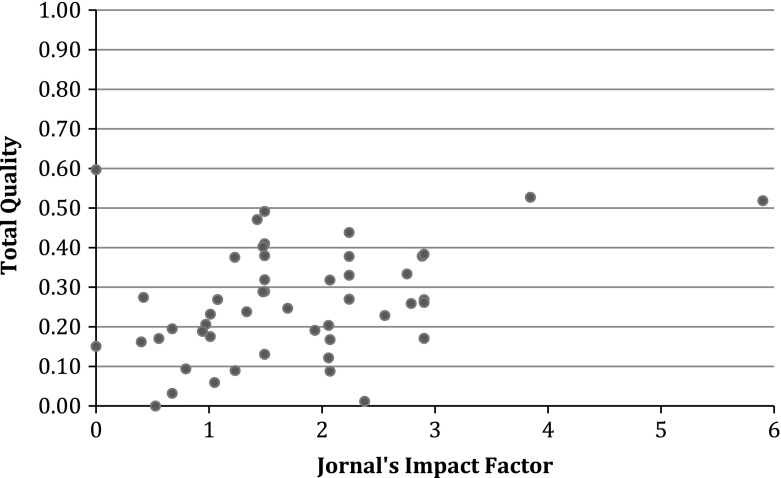
Total quality of research papers and journal impact factors

To analyze more, journal impact factors and aggregated quality of research papers are plotted for all research papers in two sections of Technology, Policy and Management faculty in Delft University of Technology (Fig. 2). As can be seen in this Figure there is no trade-offs between quality and impact factor.

## Conclusion

Research assessment is one of the challenging problems that scientists have faced and there is no standard and unique solution for that. To overcome the deficiencies of peer review, some other alternatives are used for research evaluation in the literature such as citation analyses. However, peer review and citation analysis do not cover completely the scientific impact of research papers. Therefore we need more quality metrics to evaluate the paper based on other aspects of quality such as, educational impact and scientific collaboration impact of the research papers.

In this study, we collected data from Scopus for 54 research papers in faculty of Technology, Policy and Management of Delft University of Technology. We measured the quality of each research paper based on the data we collected from Scopus and weights of each metrics from the strategic point of view of faculty.

Our study contributes to the ongoing debate of measuring the quality of scientific research outputs. In overall, this study contributes to the research evaluation literature in three ways: in this study (1) we tried to find objective metrics (citation, engagement, scientific collaboration and educational metrics) as much as possible rather than relying merely on peer review criterion and citation analysis; (2) not only we found these objective metrics with their items, we emphasized that considering the importance and weights of these metrics are essential; (3) we found that impact factor of journal is not a suitable metric to measure the quality of research papers.

Based on our results, one main policy which can be used by research institutes and scientific policy makers is to motivate researchers (authors) to be active in using and developing their social networks as much as possible and introduce their works in these online networks.

In this study we evaluated the importance of quality metrics in strategic level of university (we collected data from the dean of faculty). However, it is worth-mentioning that all metrics have not the same value and importance in different levels of assessment, as each level may have different mission. Hence, one interesting future research in this area is evaluation of the identified metrics in both levels of individual (quality assessment by researchers) and strategic (quality assessment by decision/policy makers in strategic level of university such as head of section or dean of faculty) and make a comparison study between self-assessment and strategic assessment.

By knowing the weight of each metric in both individual and strategic levels of university, it is possible to compare the quality of scientific outputs from different perspectives. Comparing self-assessment with strategic assessment provides a situation for researchers to be more in line with the mission of university. At the same time policy makers at university can modify their policies/decisions based on the results of self-assessment.

In this study we assessed the quality of research papers in one faculty. However, it would be interesting to consider the effect of different values and importance of metrics on the final quality of research papers in different faculties. This issue may influence the level of final quality of two research papers with the same quality score but with different importance level of different metrics in different faculties. More precisely, one metric may have more importance in one faculty compared to the other faculties. Therefore, this differences impact on the final quality of two research papers with the same score.

## Footnotes

1
www.arwu.org.

2
www.timeshighereducation.co.uk/world-university-rankings/.

3
www.scimagoir.com.

4
www.snowballmetrics.com.

5
www.scopus.com.

6 Transport and Logistic section and Economics of Technology and Innovation section in Delft University of Technology (the two sections belong to the faculty of Technology, Policy and Management; from the dean of this faculty we have found the weights of the assessment criteria). Based on Scopus, the researches which are done in these two sections are categorized in social sciences.
